# Assessing Racial Heterogeneity in “Housing First” Supports’ Effectiveness Among Older Adults Experiencing Homelessness: Evidence From Los Angeles County

**DOI:** 10.1093/geront/gnaf050

**Published:** 2025-02-06

**Authors:** Jared N Schachner, Steven Schmidt, Gary D Painter

**Affiliations:** Price School of Public Policy, University of Southern California, Los Angeles, California, USA; Department of Sociology, University of Southern California, Los Angeles, California, USA; Carl H. Lindner College of Business, University of Cincinnati, Cincinnati, Ohio, USA

**Keywords:** “Housing First” interventions, Housing insecurity/homelessness, Older adults of color

## Abstract

**Background and Objectives:**

Just as the U.S. population is aging and racially diversifying, housing prices are spiking. These conditions could spur a homelessness crisis among older adults of color. Although researchers have examined racial stratification and age-based differences in homelessness risk, separately, few studies probe whether age and race interact to render older adults of color more vulnerable to repeated episodes of homelessness than younger adults and older White adults. Fewer still have considered whether and why “Housing First” interventions (e.g., rapid rehousing [RRH], permanent supportive housing [PSH]) are disproportionately effective—or ineffective—in reducing this risk for older adults of color.

**Research Design and Methods:**

Using individual-level data from Los Angeles County’s Homelessness Management Information System, tracking Continuum of Care (CoC) services received between 2013 and 2019, we show how race and age jointly shape the risk of receiving additional CoC homelessness services after an initial CoC service. Linear probability models reveal whether PSH and RRH disproportionately reduce this risk for Black versus White older adults.

**Results:**

Our analyses indicate an age-race interaction, whereby Black adults aged 55–64 exhibit a particularly high risk of receiving additional CoC homelessness services after an initial service. PSH disproportionately reduces this risk for Black older adults.

**Discussion and Implications:**

We conclude that “Housing First” interventions may hold particular promise for older adults of color, perhaps because these groups sort into more effective programs, on average. Future research on aging and housing should highlight heterogeneity and consider program sorting processes as a potential explanation for it.

Two major demographic trends—the rapid aging and racial diversification of the U.S. population (Frey, 2014)—have collided with spiraling housing costs to spur a looming homelessness crisis among older adults of color. By virtue of being homeless, of advanced age, and racially minoritized, this group is triply disadvantaged and likely at exceptionally high risk of adverse health outcomes, especially amidst the coronavirus disease 2019 (COVID-19) pandemic (Porter et al., 2022). Despite the growing size and acute vulnerability of older adults of color, recent studies typically examine the experiences of housing precarity among racial minorities or older adults separately (Culhane et al., 2019), rather than both simultaneously (c.f. Paul et al., 2020; Scheckler et al., 2023). In this study, we apply an intersectional lens to quantify the risk of previously unhoused older adults of color who received homelessness services subsequently returning for additional services, suggesting unresolved housing precarity—what we refer to as the *risk of returning to homelessness services* (Milburn et al., 2021). We also assess whether “Housing First” interventions, which have proven highly effective for the general population, are particularly impactful in reducing this risk for older adults of color.

Our study answers two key research questions: (1) How does the risk of returning to homelessness services vary between older (age 55+) and younger adults overall, and between Black and White older adults? (2) Are “Housing First” interventions (e.g., permanent supportive housing [PSH], rapid rehousing [RRH]) particularly effective—relative to the common emergency shelter (ES) alternative—in reducing the risk of returning to homelessness services for Black versus White older adults?

Based on prior research, we expect older Black adults to exhibit elevated risk of repeatedly returning to homelessness services. PSH may be particularly effective in reducing this risk relative to the ES alternative among older Black adult subpopulation, given the subgroup’s higher rates of vulnerabilities that these “Housing First” interventions are specifically designed to address, including health disabilities and more extensive prior homelessness histories, prompted by cumulative disadvantages, including criminal justice contact, labor market disconnection, and eviction.

We examine these possibilities using de-identified data from the Homelessness Management Information System (HMIS) in Los Angeles County, which is arguably the epicenter of America’s homelessness surge. Our data set tracks nearly all adults interfacing with the county’s Continuum of Care (CoC), a community-wide system integrating local resources, services, and data to support those experiencing homelessness in securing stable housing, from 2013 through 2019. Indicators include what types of CoC services adults received and the timeframe during which they received them. We first describe differences across age-race subgroups in rates of returning for additional CoC services after receiving a prior CoC service and find that middle-aged and older Black adults exhibit particularly high risk of returning to homelessness services. We then use multivariate models to gauge whether accessing PSH or RRH programs, as opposed to ES, reduces the risk of returning to homelessness services for the overall population and disproportionately for Black older adults. Indeed, both PSH and RRH are highly effective in reducing this risk among older and younger adults alike, but Black adults—particularly Black older adults—see the greatest risk reduction. However, our hypothesis that this heterogeneity is explained by subgroup differences in the vulnerabilities PSH and RRH are uniquely equipped to address is not supported.

Overall, the results underscore the value of applying an intersectional lens to illuminate variation in the risk of continuously returning to homelessness services and to highlight variation in particular interventions’ effectiveness in reducing this risk across subpopulations. The intersectional approach suggests “Housing First” interventions may hold particular promise for older adults of color, reflecting, perhaps, racially stratified program selection processes. Future research should highlight heterogeneity and further scrutinize group differences in program sorting patterns as a potential source of variation in intervention effectiveness.

## Housing Precarity Among People of Color and Older Adults

Recent research casts structural racism as a fundamental cause of homelessness and documents the disproportionate representation of people of color—particularly Black and Indigenous populations—among the unhoused (Fowle, 2022; Jones, 2016; Olivet et al., 2021). Scholars implicate race-based discrimination across many domains throughout the life course (e.g., via healthcare, the criminal justice system, and housing market), which fosters cumulative disadvantages that may substantially elevate homelessness risk; these disadvantages, in turn, become compounded as a consequence of homelessness (Paul et al., 2020). For example, prior criminal justice contact—which is disproportionately endured by Black Americans, stigmatizing them in labor and housing markets (DeMarco, 2023; Pettit & Western, 2004)—may contribute to elevated housing instability and homelessness risk among Black adults, relative to White ones. Further, the stigma of enduring a prior eviction or homelessness episode may interact with racial discrimination in ways that hamper unhoused people of color’s attempts to reenter the job market or acquire secure private market housing. Some evidence also suggests that health challenges are more common among certain unhoused minoritized groups, compared to unhoused White adults (Kushel et al., 2001), creating additional barriers to long-term housing stability, absent robust interventions.

Similarly, several recent studies probe the unique risk factors and consequences for older adults experiencing homelessness. Growing older comes with myriad challenges, including physical and cognitive decline, as well as death or impairment of partners, family, and friends. These conditions can foster income insecurity and social isolation and, in turn, housing insecurity, particularly after economic shocks (Brown et al., 2011, 2016; Burns & Sussman, 2019; Herbert & Molinsky, 2019). Health conditions loom large among older adults not only as a key cause of homelessness but also as a consequence of it (Brown et al., 2022). Acute health challenges, and the elevated economic and housing insecurity risks they often engender, may render older adults of all race/ethnic groups vulnerable to repeated episodes of homelessness.

### Applying an Intersectional Lens to Illuminate Homelessness Risk Among Older Adults of Color

Although these emerging lines of research constitute important steps forward, studies probing the intersection between them remain scarce. This void is particularly glaring given that stratification theories increasingly highlight the value of adopting intersectional frameworks, whereby structural vulnerabilities (e.g., age, race, and gender) are posited to compound each other in complex and highly consequential ways across the life course (Homan et al., 2021). “Intersectional disadvantage” may be particularly salient to housing and homelessness.

For example, older adults of color may exhibit elevated rates of prior criminal justice contact or prior evictions across their life course, patterns that reflect Black older adults coming of age during the mass incarceration age and also during a period when eviction protection policies and services were scarce (Neil & Sampson, 2021; Paul et al., 2020). Moreover, acute health challenges, which may hamper economic and housing security, disproportionately afflict Black older adults compared to older White adults and younger Black adults. A social determinants of health framework highlights how structural racism engenders increased rates of chronic disease and mental health challenges (e.g., via residential segregation, environmental contamination, and trauma) among people of color. Aging likely substantially amplifies these disadvantages by fostering heightened vulnerability to them (Ferraro & Shippee, 2009), a process called accelerated aging. We thus expect to find that Black older adults exhibit a higher probability of experiencing repeated episodes of homelessness than do older White adults or younger Black adults.

## The Promise of “Housing First” Interventions

If older adults of color, particularly Black older adults, exhibit elevated vulnerability to repeated episodes of homelessness, targeting this group with the most robust, evidence-based housing interventions available is warranted. In recent years, a consensus has emerged that “Housing First” programs are most promising in securing long-term housing stability for individuals at risk of homelessness—and that these programs are far more effective than common alternatives, especially ES. “Housing First” programs’ approach upends the prevailing perception in the twentieth century that people experiencing homelessness must first demonstrate “suitability” for housing (e.g., by resolving behavioral health challenges, ceasing drug use, and participating in services) before accessing housing. Instead, “Housing First” programs assume that permanent shelter is foundational, enabling residents to participate in supportive services and to resolve key health challenges. The model also prioritizes clients’ specific needs and preferences when selecting interventions; this alignment may boost the likelihood of success (Woodhall-Melnik & Dunn, 2016).

There are two primary types of “Housing First” programs. The first is RRH, which provides short-term rental assistance (e.g., in the form of time-limited vouchers) paired with select services for households enduring temporary disruptions that increase homelessness risk. These services often include support in identifying suitable housing units, rental payment and move-in cost assistance, and case management. The second program type is PSH, which provides more intensive supports centered around housing unit provision to individuals who would otherwise struggle to remain stably housed, due to serious physical and mental health concerns, including chronic illness and substance use disorders. PSH interventions can follow either a project-/site-based model—where PSH residents live together in a single complex—or a scattered-site (SS) approach, where PSH residents rent from private landlords but receive services. Participants in this intervention often have endured chronic homelessness. They are therefore expected to receive program support for a much longer duration than those who receive RRH (Henwood et al., 2013).

**Figure 1.**
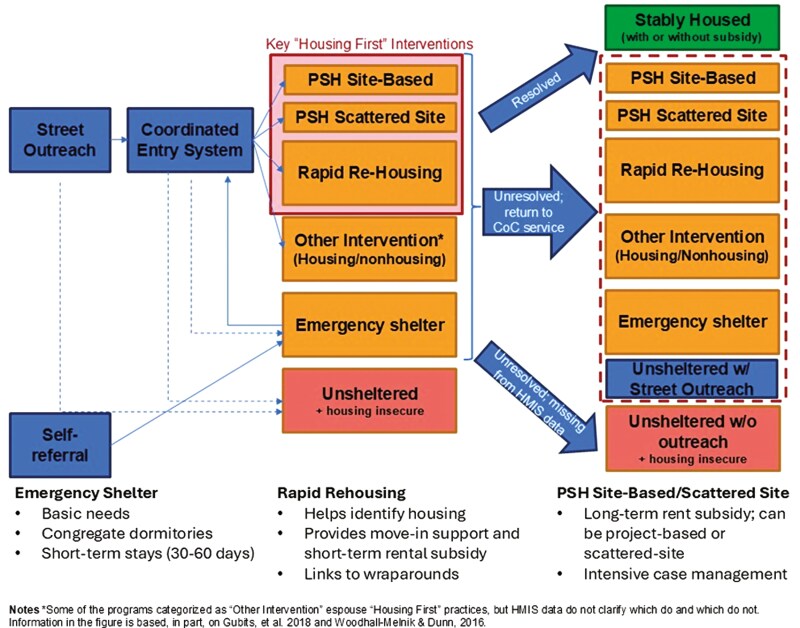
Overview of Continuum of Care (CoC) service flow. PSH = permanent supportive housing.

In recent decades, investments in “Housing First” programs like RRH and PSH have increased rapidly but geographically unevenly across U.S. CoCs. This geographic variation reflects a key feature of U.S. homelessness policy: despite the ubiquitous, U.S. Department of Housing and Urban Development-mandated CoC organizational structure, local areas are afforded substantial flexibility in deciding the jurisdictional level at which their CoCs are organized (e.g., city, county, and state), as well as how they respond to local needs and preferences. This variation is evident not only in the wide distribution of financial resources available to CoCs—and in the share of resources they each invest in “Housing First” interventions—but also in how they allocate often-scarce PSH and RRH slots to individuals at risk of homelessness within their jurisdictions. This allocation of “Housing First” program slots to individuals typically entails some form of CoC-run Coordinated Entry system whereby unsheltered individuals and emergency shelter residents are initially identified, triaged, assessed for risk in a standardized way, and then prioritized for RRH or PSH referrals once capacity allows, with the highest-risk individuals often receiving the highest priority for PSH. However, specific approaches to creating access points for those in need, assessing risk, and prioritizing individuals based on risk assessments vary widely across U.S. CoCs. Figure 1 provides a high-level overview of how a typical U.S. CoC functions in linking unsheltered and housing precarious individuals to emergency shelter, “Housing First” interventions, or other programs.

### Why Older Adults of Color in General, and Black Older Adults in Particular, May Benefit Most

Although a growing literature confirms “Housing First” interventions’ effectiveness for the population of housing insecure individuals as a whole (Gubits et al., 2018; Woodhall-Melnik & Dunn, 2016), surprisingly little research has scrutinized whether and why these programs are disproportionately effective—or ineffective—relative to common alternatives, like emergency shelter, for various demographic subgroups. This omission is important because the population at risk of homelessness is highly, perhaps increasingly, heterogeneous along various axes, including age and race. Much as the emerging paradigm of “precision medicine” highlights the potential power of customizing treatments for health conditions across subgroups, the overall (i.e., “main”) effects of “Housing First” interventions may obscure substantial variation.

Clarifying whether these programs are particularly effective or ineffective for older adults in general, and Black older adults, in particular, given these groups’ growing size and acute vulnerabilities, is crucial. But doing so requires first clarifying what the counterfactual condition is. In the absence of PSH or RRH access, individuals at risk of homelessness are often in emergency shelters/ES—a low-intensity, short-duration intervention that is frequently used in U.S. CoCs.

Compared to White older adults, Black older adults may disproportionately benefit from PSH versus ES both because (1) ES is particularly *ineffective* for individuals with prior criminal justice experiences, extensive histories of housing instability, and complex sets of needs (e.g., physical and mental disabilities; Quirouette, 2016) each of which is particularly common among older Black adults and because (2) PSH’s intensive support services model renders it particularly *effective* for individuals with some of these same risk factors, especially physical and mental conditions, as well as social isolation (Henwood et al., 2015; Padgett et al., 2020; Rog et al., 2014).

Black older adults may also see disproportionate benefits from RRH versus ES compared to White older adults, though for distinct reasons. Even if they are healthy, Black older adults at risk of homelessness may face uniquely formidable barriers to securing stable housing in the private market; they may be more likely to need both the financial support and housing market services RRH provides. Moreover, this subpopulation exhibits elevated rates of prior criminal justice contact and eviction proceedings (Graetz et al., 2023; Pettit & Western, 2004) and extensive research suggests some landlords use these records as a pretext to reject prospective tenants. Others discriminate on the basis of race or age, regardless of criminal justice and housing histories, despite fair housing laws (Gaddis & DiRago, 2023).

Overall then, we expect “Housing First” interventions to be significantly more effective in reducing the risk of experiencing repeated homelessness episodes relative to the emergency shelter counterfactual for Black older adults compared to White ones. Despite these potentially amplified benefits, evidence suggests Black adults in general—and Black older adults in particular—may have a diminished chance of accessing them. This disparity in access could reflect key features of the PSH and RRH assignment process employed by some CoCs' coordinated entry systems. For example, a common risk assessment tool used to prioritize individuals for “Housing First” interventions, known as VI-SPDAT, has been shown to systematically rate White individuals as higher risk and therefore higher priority for scarce “Housing First” slots (Cronley, 2022). It is also possible that even among the subset of individuals who are successfully referred to these slots, there are racial gaps in the quality of PSH and RRH programs they access. Minimal research to date has probed these possibilities.

## Data and Methods

To empirically examine our expectations of heterogeneity in risk and “Housing First” program effectiveness, we use the Research Accelerator data set (a de-identified extract of HMIS data hosted at the California Policy Lab), which tracks individuals interfacing with Los Angeles County’s CoC from 2010 on. By virtue of its expansive size and time horizon, as well as its identification of service types, this data set is uniquely equipped for examining heterogeneity in risk and interventions’ effectiveness. Los Angeles is a strategic case insofar as it is widely perceived to be on the front lines of America’s homelessness surge, due in part to its high levels of poverty, inequality, and housing costs (Colburn & Aldern, 2022; Sampson et al., 2017; Schachner & Sampson, 2020). The population served by the Los Angeles County CoC is also exceptionally large and diverse, permitting valid estimates of age- and race-stratified risk and of heterogeneous program effects by subgroups. Moreover, Los Angeles is home to a large, ever-expanding set of “Housing First” programs.

### Analytic Sample

Our analytic sample begins with all HMIS-reported Los Angeles County CoC service contacts that started between 2013 and 2019 (*N* = 541,831). We limit our sample to CoC service contacts linked to adults (i.e., age 25+, so transition-aged youth are excluded) at baseline (2013) (Service *N* = 363,484, Adult *N* = 168,340). Of these, we drop 54,788 CoC service contact records that indicated a mix of temporally overlapping service types (e.g., emergency shelter, rapid rehousing, permanent supportive housing, transitional housing) and another 24,432 CoC service contacts that either began less than a week after the entry date or exit date (if applicable; whichever is later) or that contained a CoC service entry date within a week of the end of our study period (December 31, 2019). These specifications mitigate bias induced by high-frequency churning (e.g., moving into and out of a given emergency shelter during a single week). Also, to partially mitigate mortality-induced attrition bias, we remove an additional 1,915 CoC service contacts marked in HMIS as ending with the death of the service recipient, as well as 28,288 service contacts that had no post-service destination data, raising the possibility that a death occurred during service. Our final analytic sample includes 254,061 CoC service “spells” nested within 155,558 unique adults. For detailed information on, and justification for, our analytic sample specification decisions, see Supplementary Material: Methodological Appendix.

### Outcome Variable

Following Milburn et al. (2021), which uses the same HMIS data set as we do, our primary outcome is a proxy for the *risk of returning to homelessness services*, operationalized as a binary indicator of whether a given individual CoC service recipient returned for an additional CoC service, of any type, after the focal CoC service spell and before the end of 2019. This outcome assumes that a given CoC service prevented an individual from returning to homelessness services (and is marked as “0”) if the said spell: (a) ends with the recipient “exiting” service and no subsequent CoC service spell occurs before December 31, 2019, or (b) is never marked as ending and the HMIS contains no additional CoC service for the individual between the service entry date and December 31, 2019.

This outcome comes with some nontrivial limitations, insofar as the risk of returning to homelessness services may not perfectly overlap with the risk of returning to homelessness in general. For example, some individuals marked as nonreturns in our data set may have returned to unsheltered homelessness and were not contacted by street outreach; others may have relocated to other CoCs. Both types of returns to homelessness would not show up in our data. These concerns are partially allayed because locating to other CoCs is less likely in Los Angeles County given its expansiveness and because individuals that return to unsheltered homelessness are likely to eventually be contacted by street outreach—contacts that are tracked in our HMIS data.

Moreover, we believe our outcome operationalization is preferable to other options available in the data. Although using service provider-coded exit destination codes that specify where exiting individuals ended up post-service may seem preferable, a sizable body of research suggests that service providers do not consistently and accurately input this information into the HMIS database (Meyer et al., 2023). Our outcome variable sidesteps these concerns because it is not reliant on service providers accurately entering exit destination information or dates, though we do use the destination codes for robustness checks, described below.

### Key Stratifying and Predictor Variables

Reflecting our intersectional lens, we stratify our analyses by *age* and *race/ethnic identity* of CoC service recipients. For age, we divide the sample of adults who have experienced homelessness into four categories based on individuals’ estimated age at baseline (i.e., year 2013), using their HMIS-entered birthdates: younger adult (age 25–44); middle-aged adult (45–54); older adult (55–64); and retirement-age adult (65+). For race/ethnicity, we use HMIS-provided identifiers: non-Hispanic White (reference group), Hispanic, Black, Asian/Pacific Islander, Native American, and Multiracial/Other. The latter four groups only include non-Hispanic individuals. We focus, in particular, on the experiences of non-Hispanic Black and non-Hispanic White individuals because they represent two of the largest race/ethnic groups engaging with homelessness services in Los Angeles County and because recent research calls for greater attention to the relationship between anti-Blackness and homelessness. Moreover, our data set contains considerably smaller numbers of non-Hispanic Asian/Pacific Islanders and Native Americans.

Our key predictors of returning to homelessness services are binary variables indicating whether the individual received one of the following *CoC service types* during a given service spell: PSH; RRH; other, housing (OTH-H: Transitional housing; Permanent housing: housing only; Permanent housing: with services but no disability required); other, non-housing interventions (OTH-N: Coordinated entry; Homelessness prevention; Services only; Street outreach; Other). The reference/counterfactual group includes all individuals who received ES. CoC service spells that entailed receiving PSH, RRH, OTH-H, or ES in combination with a service in the OTH-N category were marked as receiving PSH, RRH, OTH-H, or ES.

To partially mitigate concerns that the estimated benefits of RRH or PSH are inflated by selection bias, we also include an extensive slate of control variables encompassing the timing of individuals’ focal CoC service spells; their gender; household structure; veteran status; disability status; and prior experiences with homelessness. For more information on these variables, see Supplementary Material: Methodological Appendix. Table 1 presents descriptive statistics for the full sample, and then stratifies by age bracket and focal racial group (i.e., Black vs White). Although Black and White adults in the analytic sample diverge vis-à-vis our outcome of interest, rates of disability and prior homelessness experiences are very similar across races within each age group.

**Table 1.** Descriptive Statistics for Los Angeles County Continuum of Care Services Received by Adults in 2013–2019

Age group (2013)^a^	All ages	Younger adult (25–44)		Middle-aged adult (45–54)		Older adult (55–64)		Retirement age (65+)	
Race/ethnicity^b^	All Races	NH White	NH Black	NH White	NH Black	NH White	NH Black	NH White	NH Black
Returned for additional CoC service	0.41 (0.49)	0.37 (0.48)	0.43 (0.49)	0.44 (0.50)	0.51 (0.50)	0.42 (0.49)	0.50 (0.50)	0.34 (0.47)	0.43 (0.50)
*Focal CoC service: type of service received*									
ES: Emergency shelter	0.38 (0.49)	0.36 (0.48)	0.40 (0.49)	0.37 (0.48)	0.43 (0.49)	0.39 (0.49)	0.42 (0.49)	0.40 (0.49)	0.43 (0.49)
PSH: Permanent supportive housing	0.02 (0.14)	0.01 (0.12)	0.02 (0.14)	0.02 (0.14)	0.03 (0.16)	0.02 (0.15)	0.03 (0.16)	0.02 (0.12)	0.02 (0.14)
RRH: Rapid rehousing	0.10 (0.30)	0.07 (0.26)	0.14 (0.35)	0.06 (0.24)	0.09 (0.29)	0.09 (0.28)	0.11 (0.31)	0.12 (0.33)	0.13 (0.33)
OTH-H: Other, housing^c^	0.04 (0.19)	0.04 (0.21)	0.03 (0.17)	0.04 (0.20)	0.04 (0.20)	0.06 (0.23)	0.05 (0.22)	0.06 (0.23)	0.06 (0.23)
OTH-N: Other, non-housing^d^	0.46 (0.50)	0.52 (0.50)	0.41 (0.49)	0.50 (0.50)	0.41 (0.49)	0.44 (0.50)	0.39 (0.49)	0.40 (0.49)	0.37 (0.48)
*Client gender/household structure*									
Gender: Man	0.63 (0.48)	0.64 (0.48)	0.55 (0.50)	0.67 (0.47)	0.66 (0.47)	0.72 (0.45)	0.75 (0.43)	0.72 (0.45)	0.76 (0.43)
Gender: Woman	0.35 (0.48)	0.35 (0.48)	0.44 (0.50)	0.32 (0.47)	0.33 (0.47)	0.27 (0.45)	0.25 (0.43)	0.28 (0.45)	0.24 (0.43)
Gender: Trans/Other	0.01 (0.07)	0.01 (0.08)	0.01 (0.09)	0.00 (0.06)	0.00 (0.06)	0.00 (0.04)	0.00 (0.04)	0.00 (0.05)	0.00 (0.00)
Gender: Unknown	0.02 (0.14)	0.00 (0.03)	0.00 (0.02)	0.00 (0.02)	0.00 (0.02)	0.00 (0.03)	0.00 (0.02)	0.00 (0.02)	0.00 (0.00)
2+ person household	0.10 (0.30)	0.07 (0.26)	0.17 (0.38)	0.03 (0.17)	0.05 (0.22)	0.03 (0.16)	0.03 (0.17)	0.03 (0.18)	0.04 (0.21)
*Client vulnerabilities and prior experiences*									
Veteran	0.13 (0.34)	0.11 (0.32)	0.08 (0.28)	0.16 (0.37)	0.16 (0.36)	0.27 (0.44)	0.28 (0.45)	0.40 (0.49)	0.36 (0.48)
Nonveteran	0.82 (0.39)	0.84 (0.37)	0.89 (0.31)	0.80 (0.40)	0.82 (0.38)	0.70 (0.46)	0.70 (0.46)	0.58 (0.49)	0.62 (0.49)
Vet status unknown	0.05 (0.22)	0.05 (0.21)	0.03 (0.16)	0.03 (0.18)	0.02 (0.15)	0.03 (0.17)	0.02 (0.13)	0.02 (0.16)	0.02 (0.14)
Disabilities									
Physical	0.24 (0.43)	0.17 (0.38)	0.16 (0.37)	0.32 (0.46)	0.31 (0.46)	0.39 (0.49)	0.41 (0.49)	0.44 (0.50)	0.45 (0.50)
Developmental	0.09 (0.28)	0.12 (0.32)	0.10 (0.30)	0.10 (0.30)	0.10 (0.30)	0.08 (0.27)	0.09 (0.28)	0.05 (0.22)	0.05 (0.21)
Chronic	0.23 (0.42)	0.18 (0.39)	0.17 (0.38)	0.29 (0.45)	0.28 (0.45)	0.36 (0.48)	0.36 (0.48)	0.38 (0.49)	0.39 (0.49)
HIV/AIDS	0.02 (0.14)	0.02 (0.15)	0.03 (0.16)	0.02 (0.15)	0.03 (0.16)	0.01 (0.11)	0.02 (0.14)	0.01 (0.11)	0.01 (0.10)
Mental	0.31 (0.46)	0.34 (0.47)	0.32 (0.47)	0.36 (0.48)	0.37 (0.48)	0.33 (0.47)	0.34 (0.47)	0.23 (0.42)	0.22 (0.41)
Substance abuse	0.05 (0.21)	0.05 (0.21)	0.03 (0.17)	0.07 (0.26)	0.05 (0.21)	0.09 (0.29)	0.05 (0.22)	0.05 (0.22)	0.04 (0.19)
# prior CoC services	1.80 (1.42)	1.68 (1.25)	1.82 (1.44)	1.84 (1.39)	2.10 (1.71)	1.78 (1.37)	2.08 (1.68)	1.56 (1.09)	1.87 (1.53)
*Times homeless in prior 3 years*									
N/A (none)	0.15 (0.35)	0.16 (0.37)	0.15 (0.35)	0.15 (0.35)	0.13 (0.33)	0.12 (0.33)	0.12 (0.32)	0.13 (0.34)	0.11 (0.31)
Once	0.24 (0.43)	0.25 (0.43)	0.24 (0.43)	0.25 (0.44)	0.22 (0.42)	0.25 (0.43)	0.21 (0.41)	0.29 (0.45)	0.22 (0.41)
Twice	0.09 (0.28)	0.08 (0.27)	0.10 (0.30)	0.08 (0.27)	0.09 (0.28)	0.08 (0.27)	0.09 (0.28)	0.06 (0.24)	0.08 (0.28)
Three times	0.05 (0.21)	0.05 (0.21)	0.05 (0.23)	0.04 (0.19)	0.05 (0.22)	0.04 (0.20)	0.05 (0.22)	0.04 (0.19)	0.05 (0.22)
Four or more times	0.14 (0.35)	0.14 (0.35)	0.15 (0.35)	0.16 (0.36)	0.17 (0.37)	0.15 (0.35)	0.15 (0.36)	0.11 (0.32)	0.13 (0.33)
Unknown	0.35 (0.48)	0.33 (0.47)	0.32 (0.47)	0.34 (0.47)	0.36 (0.48)	0.37 (0.48)	0.39 (0.49)	0.38 (0.48)	0.42 (0.49)
Person *N*	155,558	17,368	29,319	11,257	17,662	6,586	11,093	1,942	2,445
Service *N*	254,061	26,767	48,760	19,233	33,618	10,785	20,934	2,830	4,131

*Notes*: CoC= continuum of care; ES = emergency shelter; HIV = human immunodeficiency virus; N/A = not available; NH = non-Hispanic; OTH-H = other-housing; OTH-N = other-nonhousing; PSH = permanent supportive housing; RRH = rapid rehousing.

^a^Analytic sample individuals’ age is estimated in the year 2013, based on their year of birth.

^b^NH White and NH Black stand for non-Hispanic White and non-Hispanic Black.

^c^Type of CoC service-Other, housing (OTH-H) includes: Transitional Housing; Permanent Housing: Housing Only; Permanent Housing: with services but no disability required.

^d^Type of CoC service-Other, non-housing (OTH-N) includes: Coordinated entry; Homelessness Prevention; Services Only; Street Outreach; Other.

### Analytic Strategy

We first descriptively compare how the risk of receiving another CoC service between 2013 and 2019 after receiving a prior service varies between White and Black adults in our sample, within each of our four age categories. Next, we run linear probability models (LPMs), using ordinary least squares, that predict this risk in a multivariate framework. We opt for LPMs over event history models for two key reasons. First, the substantive interpretation of coefficients generated by LPMs is much more straightforward and intuitive than is the interpretation of hazard ratios generated by event history models; effect magnitudes can be compared across stratified models for the former but not for the latter, and most of our models below are age-stratified. Second, our core parameters of interest (described below) are interaction terms, which present inference challenges when using nonlinear model specifications. An emerging consensus suggests interaction terms’ magnitude and standard errors estimated from nonlinear models are not readily interpretable the way interactions from linear models are (Long & Mustillo, 2018; Mize, 2019; Mustillo et al., 2018). However, in robustness checks, we replicate key model specifications below using the event history model framework. The core findings remain substantively unchanged.

The first sets of LPMs gauge age-based heterogeneity in the effect of receiving “Housing First” CoC services (i.e., PSH, RRH) versus emergency shelter on the risk of returning to homelessness services:


$$\begin{array}{lllll} & {\left(Received\;Additional\;CoC\;Service\;After\;Focal\;CoC\;Service\right)}_{ij}=\; & \;\beta_{0}+\beta_{1}{\left(PSH\right)}_{ij}+\beta_{2}{\left(RRH\right)}_{ij}+\beta_{3}{\left(OTH-H\right)}_{ij}\; & +\beta_{4}{\left(OTH-N\right)}_{ij}+\beta_{5}{\left(Age\;45-54\right)}_{j}\; & +\beta_{6}{\left(55-64\right)}_{j}+\beta_{7}{\left(65+\right)}_{j}+\;\ldots +e_{ij} \end{array}$$

The binary outcome, which captures whether individual *j* returns to homelessness services after prior CoC service spell *i,* is predicted as a function of: the type of CoC intervention received and age bracket, as well as the individual’s continuous age in 2013 and age-squared and fixed effects capturing CoC service entry timing. Our inclusion of continuous age and age-squared controls, in addition to age brackets, reflects concerns that our four age brackets are sufficiently large that other predictors’ effects may be confounded by age gaps *within* the stratum and that intra-stratum age differences may exhibit a nonlinear relationship to our outcome.

Subsequent models include interactions between the CoC service type and age bracket fixed effects (twelve total). These interaction terms’ coefficients are the focal parameters of interest, capturing how much stronger or weaker the effects of receiving PSH and RRH are on the probability of returning to homelessness services (vs receiving ES), for older adults relative to younger adults (age 25–44).

We then stratify the sample across our four age groups (25–44, 45–54, 55–64, 65+) to assess whether there is evidence not only of age-based heterogeneity in RRH and PSH effectiveness but also racial heterogeneity in these programs’ effectiveness *within each age bracket*. For more details on the equation we use for these age-stratified models, see Supplementary Material: Methodological Appendix.

It is important to note that all of our LPMs cluster standard errors by individual because approximately a third of individuals in our analytic sample contributed multiple CoC service spells to our analytic sample. We replicated key model specifications using two-level hierarchical linear models (HLMs; level-1: CoC service spell; level-2: individual), which are specifically designed for nested data like ours and adjust standard errors accordingly. HLM results, reported below, are virtually indistinguishable from those generated by our LPMs with clustered standard errors.

## Results

Across our full analytic sample, the risk of returning to homelessness services exhibits a nonlinear age gradient, with middle-aged and older adults not yet of retirement age exhibiting the highest risk. Within these two groups, ~45% of service spells were followed by a subsequent CoC service. For younger adults and retirement-age adults, the equivalent estimates are 38% and 37%, respectively.

The top row of Table 1 suggests that, congruent with prior studies, Black individuals are more likely to return to homelessness services than are White ones. Black adults within each age category exhibit a 6–9 percentage point (pp) elevated risk of post-CoC service return for additional services compared to White individuals. The Black–White gap in risk grows across the age distribution; it is smallest among young adults and nearly doubles among retirement-age adults. Given Black older adults’ disproportionate vulnerability, it is critical to clarify what interventions serve them most effectively.

Perhaps surprisingly, Hispanic adults’ risk of returning to CoC services closely mirrors that of Whites within each of our four age strata (see Supplementary Figure S1, Panel A). However, this risk may be underestimated due to Hispanics’ higher rates of undocumented status and lower rates of institutional trust. These factors may lead Hispanic individuals to disproportionately opt for unsheltered homelessness and avoid CoC touchpoints, including street outreach (Aiken et al., 2021).

Before reporting our multivariate model results, we first descriptively assess whether individuals of any age or race who receive PSH or RRH exhibit a lower likelihood of returning for CoC services compared to those in ES. The unadjusted patterns (Table 2) reinforce the large literature establishing that “Housing First” interventions are much more effective than ES in preventing repeated homelessness episodes (Gubits et al., 2018). For the full analytic sample, receiving “Housing First” interventions like PSH and RRH predicts an unadjusted ~20 percentage point (pp) reduction in the risk of returning to CoC services relative to receiving ES. Congruent with Milburn et al. (2021), Table 2A suggests that among PSH recipients, Black adults exhibit elevated risk of subsequently returning to CoC service relative to White adults. However, when this risk is benchmarked against same-race adults in ES—our key comparison of interest—the unadjusted PSH-associated drop is steeper for Black than White adults in both absolute terms (17 pp vs 13 pp) and in relative terms (30% vs 27%). Stratifying by age, Table 2B shows that the Black versus White difference in PSH-associated drops in risk are largest within the two oldest groups in both absolute and relative terms (55–64: 24 pp vs 16 pp, 39% vs 33%; 65+: 29 pp vs 12 pp, 55% vs 29%). Yet Table 1 suggests only a small proportion of CoC service spells within our analytic sample are classified as PSH (0.02) or RRH (0.10). More than a third of services are classified as ES.

**Table 2.** Descriptive Statistics: Probability of Returning for Additional Los Angeles County CoC Service after Receiving a CoC Service (2013–2019) by Age, Race, and CoC Service Type

A. By race (all ages^a^ pooled)								
Race/ethnicity^b^	All races				NH White		NH Black	
Overall	0.41 (0.49)				0.40 (0.49)		0.47 (0.50)	
*Focal CoC service: type of service received*								
ES: Emergency shelter	0.52 (0.50)				0.48 (0.50)		0.56 (0.50)	
PSH: Permanent supportive housing	0.36 (0.48)				0.35 (0.48)		0.39 (0.49)	
RRH: Rapid rehousing	0.28 (0.45)				0.27 (0.45)		0.30 (0.46)	
OTH-H: Other, housing^c^	0.44 (0.50)				0.40 (0.49)		0.50 (0.50)	
OTH-N: Other, non-housing^d^	0.36 (0.48)				0.37 (0.48)		0.41 (0.49)	
Person *N*	155,558				37,153		60,519	
Service *N*	254,061				59,615		107,443	

B. By race and age								
Age group (2013)	Younger adult (25–44)		Middle-aged adult (45–54)		Older adult (55–64)		Retirement age (65+)	
Race/ethnicity	NH White	NH Black	NH White	NH Black	NH White	NH Black	NH White	NH Black
Overall	0.37 (0.48)	0.43 (0.49)	0.44 (0.50)	0.51 (0.50)	0.42 (0.49)	0.50 (0.50)	0.34 (0.47)	0.43 (0.50)
*Focal CoC service: type of service received*								
ES	0.45 (0.50)	0.52 (0.50)	0.52 (0.50)	0.60 (0.49)	0.49 (0.50)	0.61 (0.49)	0.42 (0.49)	0.53 (0.50)
PSH	0.31 (0.46)	0.40 (0.49)	0.40 (0.49)	0.41 (0.49)	0.33 (0.47)	0.37 (0.48)	0.30 (0.46)	0.24 (0.43)
RRH	0.26 (0.44)	0.29 (0.45)	0.32 (0.47)	0.32 (0.47)	0.26 (0.44)	0.31 (0.46)	0.21 (0.41)	0.26 (0.44)
OTH-H	0.35 (0.48)	0.48 (0.50)	0.48 (0.50)	0.51 (0.50)	0.40 (0.49)	0.52 (0.50)	0.34 (0.47)	0.43 (0.50)
OTH-N	0.34 (0.47)	0.38 (0.48)	0.40 (0.49)	0.45 (0.50)	0.39 (0.49)	0.44 (0.50)	0.30 (0.46)	0.39 (0.49)
Person *N*	17,368	29,319	11,257	17,662	6,586	11,093	1,942	2,445
Service *N*	26,767	48,760	19,233	33,618	10,785	20,934	2,830	4,131

*Notes*: CoC= continuum of care; ES = emergency shelter; NH = non-Hispanic; OTH-H = other-housing; OTH-N = other-nonhousing; PSH = permanent supportive housing; RRH = rapid rehousing.

^a^Analytic sample individuals’ age is estimated in the year 2013, based on their year of birth.

^b^NH White and NH Black stand for non-Hispanic White and non-Hispanic Black.

^c^Type of CoC service-Other, housing (OTH-H) includes: Transitional Housing; Permanent Housing: Housing Only; Permanent Housing: with services but no disability required.

^d^Type of CoC service-Other, non-housing (OTH-N) includes: Coordinated entry; Homelessness Prevention; Services Only; Street Outreach; Other.

### Multivariate Models: Age Heterogeneity

Next, we specify multivariate models predicting whether a given CoC service spell was followed by a subsequent return to additional CoC services. Our first LPM (Table 3; Model 1) pools service spell observations from all age groups together and captures racial differences in the probability of returning to CoC service, with a small set of controls included. Congruent with the descriptive patterns reported above, when comparing adults (age 25+) of all ages, Black individuals exhibit a higher likelihood (3 pp) of returning to homelessness services post-CoC service compared to White ones. Black disadvantage remains detectable when age, gender, and household structure control variables are included (Model 2). This model also confirms the nonlinear relationship between age and risk: middle-age (45–54) and older adults (55–64) are each 2 pp more likely to return to homelessness services than are younger adults (25–44), but retirement-age adults are not at significantly higher risk.

**Table 3.** Linear Probability Models (Ordinary Least Squares) Predicting Probability of Returning for Additional Los Angeles County CoC Service After Receiving a CoC Service (2013–2019) (*N* = 254,061, Person *N* = 155,558)

	Model 1	Model 2	Model 3	Model 4	Model 5	Model 6
*Race/ethnicity* (ref: non-Hispanic White; *all groups are non-Hispanic, unless noted*)						
Black	0.03** (0.00)	0.04** (0.00)	0.04** (0.00)	0.04** (0.00)	0.04** (0.00)	0.04** (0.00)
Hispanic	−0.03** (0.00)	−0.01** (0.00)	−0.01** (0.00)	−0.01* (0.00)	−0.01** (0.00)	−0.01* (0.00)
Asian/Pacific Islander	−0.04** (0.01)	−0.03** (0.01)	−0.03* (0.01)	−0.03** (0.01)	−0.03** (0.01)	−0.03** (0.01)
Native American	0.06** (0.01)	0.06** (0.01)	0.06** (0.01)	0.05** (0.01)	0.06** (0.01)	0.05** (0.01)
Multiracial/Other	−0.17** (0.01)	−0.04** (0.01)	−0.04** (0.01)	−0.01 (0.01)	−0.04** (0.01)	−0.01 (0.01)
*Age category* (as of 2013; ref: 25–44)						
45–54		0.02** (0.00)	0.02** (0.00)	0.02** (0.00)	0.03** (0.01)	0.03** (0.00)
55–64		0.02** (0.01)	0.02** (0.01)	0.02** (0.01)	0.04** (0.01)	0.03** (0.01)
65+		0.01 (0.01)	0.01 (0.01)	0.01 (0.01)	0.01 (0.01)	0.01 (0.01)
*Focal CoC service: type of service received^a^* (reference group: ES) *and age–service type interactions*						
PSH			−0.18** (0.00)	−0.19** (0.01)	−0.16** (0.01)	−0.13** (0.03)
RRH			−0.19** (0.01)	−0.16** (0.00)	−0.15** (0.00)	−0.07** (0.01)
OTH-H			−0.10** (0.01)	−0.08** (0.01)	−0.11** (0.01)	−0.11** (0.02)
OTH-N			−0.07** (0.00)	−0.02** (0.00)	−0.06** (0.00)	−0.04** (0.01)
PSH × 45–54					−0.04* (0.02)	−0.03^+^ (0.02)
PSH × 55–64					−0.07** (0.02)	−0.05* (0.02)
PSH × 65+					−0.09** (0.03)	−0.06^+^ (0.03)
RRH × 45–54					−0.06**(0.01)	−0.05** (0.01)
RRH × 55–64					−0.08** (0.01)	−0.07** (0.01)
RRH × 65+					−0.03* (0.02)	−0.01 (0.02)
OTH-H × 45–54					0.02 (0.01)	0.01 (0.01)
OTH-H × 55–64					0.01 (0.01)	−0.01 (0.01)
OTH-H × 65+					0.01 (0.02)	−0.01 (0.02)
OTH-N × 45–54					−0.01 (0.00)	−0.01^+^ (0.00)
OTH-N × 55–54					−0.01* (0.01)	−0.01* (0.01)
OTH-N × 65+					0.01 (0.01)	0.01 (0.01)
*Gender and household structure*						
Gender: Woman		0.02** (0.00)	0.02** (0.00)	0.02** (0.00)	0.02** (0.00)	0.02** (0.00)
Gender: Trans/Other		0.08** (0.01)	0.07** (0.01)	0.06** (0.01)	0.08** (0.01)	0.06** (0.01)
Gender: Unknown		−0.30** (0.01)	−0.29** (0.01)	−0.11** (0.01)	−0.29** (0.01)	−0.11** (0.01)
2+ person household		−0.14** (0.00)	−0.08** (0.00)	−0.06** (0.00)	−0.09** (0.00)	−0.07** (0.00)
*Vulnerabilities and prior experiences* (based on 2013–2019 data)						
Veteran				0.01** (0.00)		0.03** (0.00)
Vet status unknown				−0.21** (0.00)		−0.24** (0.01)
Physical disability				0.01** (0.00)		0.01** (0.00)
Developmental disability				0.01** (0.00)		0.01* (0.01)
Chronic disability				−0.01* (0.00)		−0.01^+^ (0.00)
HIV/AIDS				0.02** (0.01)		0.03** (0.01)
Mental disability				0.03** (0.00)		0.04** (0.00)
Substance abuse				0.00 (0.00)		−0.00 (0.01)
# prior CoC services				0.13** (0.00)		0.12** (0.00)
# prior CoC services-squared				−0.01** (0.00)		−0.01** (0.00)
Times homeless in prior 3 years (ref: none, N/A)						
Once				0.06** (0.00)		0.05** (0.01)
Twice				0.07** (0.00)		0.06** (0.01)
Three times				0.06** (0.01)		0.05** (0.01)
Four or more times				0.06** (0.00)		0.06** (0.01)
Unknown				0.04** (0.00)		0.06** (0.01)
Vulnerabilities/past experiences × service type interactions						X

*Notes*: CoC= continuum of care; ES = emergency shelter; *N/A* = not available; OTH-H = other-housing; OTH-N = other-nonhousing; PSH= permanent supportive housing; RRH = rapid rehousing. All models include fixed effects capturing: individuals’ first year of CoC service (2013–2019); service entry date year, service entry date month, and service entry date year-month combination. Models 2–6 control for continuous age (as of 2013) and age-squared. Standard errors are clustered by person. **p < .01, *p < .05, +*p* < .10 (two-tailed test)

^a^Type of CoC service-PSH = Permanent supportive housing; RRH = Rapid rehousing; OTH-H includes = Transitional Housing; Permanent Housing: Housing Only; Permanent Housing with services no disability required. OTH-N includes: Coordinated entry; Services Only; Homelessness Prevention; Street Outreach; Other.

Model 3 adds binary indicator variables capturing CoC service type for the focal service spell. Prior research suggests PSH and RRH are highly effective. Indeed, the coefficients on these indicator variables are both significant, negative, and large in magnitude; across the full sample, receiving RRH or PSH predicts ~20 pp reduction in risk of returning to CoC services, relative to ES.

Model 4 incorporates an extensive set of variables capturing differences in individuals’ vulnerabilities and prior experiences. These variables’ coefficients confirm the expected elevated risk conferred by veteran status, many types of disability, and multiple prior homelessness experiences. But most important for this study’s purposes, the coefficients on PSH and RRH do not meaningfully attenuate, even after controlling for these acuity proxies. Further, the Black disadvantage remains detectable, suggesting Black adults are at heightened risk of returning to homelessness services, even when compared to White adults with very similar profiles.

Models 5 and 6 examine whether “Housing First” interventions like PSH and RRH are particularly effective in reducing risk among older versus younger adults, regardless of race/ethnic background. Indeed, they are. PSH’s estimated effects increase for older age brackets, with the effect amplified by ~50% for retirement-age adults compared to otherwise-similar adults age 25–44. RRH’s estimated effects are strongest for older adults ages 55–64 but are still significantly larger for retirement-age adults compared to adults ages 25–44. This pattern of age heterogeneity attenuates slightly but still remains detectable when controlling for an extensive set of individual-level vulnerability and past experience controls (Model 6). Overall, these models suggest that RRH and PSH appear extremely effective across the age distribution and particularly for older adults (55+).

### Racial Heterogeneity in “Housing First” Interventions’ Effectiveness

We next shift from age to racial heterogeneity in PSH and RRH effects, examining moderation patterns within the four age groups—focusing specifically on the two older ones. The models in Table 4 are stratified, accordingly (Models 1–4: younger adults, 25–44 as of 2013; Models 5–8: middle-age adults, 45–54; Models 9–12: older adults, 55–64; Models 13–16: retirement-age adults, 65+).

**Table 4.** Age-Stratified Linear Probability Models (Ordinary Least Squares) Predicting Probability of Returning for Additional Los Angeles CoC Service After Receiving a CoC Service (2013-2019)

	Young adult (25–44)^a^ (service *N* = 126,558; person *N* = 81,672)				Middle-age adult (45–54) (service *N* = 74,861; person *N* = 42,747)			
	Model 1	Model 2	Model 3	Model 4	Model 5	Model 6	Model 7	Model 8
*Race/ethnicity × CoC service type interactions*								
Black × PSH			−0.00 (0.03)	0.00 (0.03)			−0.09** (0.03)	−0.09** (0.03)
Black × RRH			−0.03* (0.01)	−0.03* (0.01)			−0.08** (0.02)	−0.07** (0.02)
Black × OTH-H^b^			0.07** (0.02)	0.07** (0.02)			−0.03 (0.02)	−0.02 (0.02)
Black × OTH-N^c^			−0.05** (0.01)	−0.06** (0.01)			−0.06** (0.01)	−0.06** (0.01)
*Focal CoC service: type of service received* (reference group: emergency shelter)								
Permanent supportive housing	−0.16** (0.01)	−0.17** (0.01)	−0.17** (0.02)	−0.12** (0.05)	−0.20** (0.01)	−0.20** (0.01)	−0.14** (0.02)	−0.07 (0.05)
Rapid rehousing	−0.15** (0.00)	−0.13** (0.00)	−0.14** (0.01)	−0.04 (0.02)	−0.21** (0.01)	−0.19** (0.01)	−0.16** (0.01)	−0.09** (0.03)
Other, housing	−0.10** (0.01)	−0.08** (0.01)	−0.13** (0.01)	−0.11** (0.04)	−0.10** (0.01)	−0.09** (0.01)	−0.09** (0.02)	−0.11* (0.05)
Other, non-housing	−0.07** (0.00)	−0.02** (0.00)	−0.03** (0.01)	−0.00 (0.02)	−0.06** (0.00)	−0.02** (0.00)	−0.03** (0.01)	0.02 (0.02)
*Race/ethnicity* (reference group: Non-Hispanic White)								
Non-Hispanic Black	0.04** (0.00)	0.04** (0.00)	0.06** (0.01)	0.06** (0.01)	0.03** (0.00)	0.03** (0.00)	0.07** (0.01)	0.06** (0.01)
*Controls^d^*								
Gender, household structure	X	X	X	X	X	X	X	X
Vulnerabilities/past experiences		X		X		X		X
Vulnerabilities/past × service types				X				X

	Older adult (55–64) (service *N* = 42,786; person *N* = 24,706)				Retirement-age adult (65+) (service *N* = 9,856; person *N* = 6,433)			
	Model 9	Model 10	Model 11	Model 12	Model 13	Model 14	Model 15	Model 16
*Race/ethnicity × CoC service type interactions*								
Black × PSH			−0.09* (0.04)	−0.09** (0.04)			−0.20* (0.09)	−0.18 (0.09)
Black × RRH			−0.07** (0.02)	−0.07** (0.02)			−0.06 (0.03)	−0.05 (0.04)
Black × OTH-H			0.02 (0.03)	0.01 (0.03)			−0.00 (0.05)	−0.01 (0.05)
Black × OTH-N			−0.11** (0.01)	−0.11** (0.01)			−0.04 (0.03)	−0.04 (0.03)
*Focal CoC service: type of service received* (reference group: emergency shelter)								
Permanent supportive housing	−0.22** (0.02)	−0.21** (0.02)	−0.16** (0.03)	−0.16** (0.06)	−0.23** (0.03)	−0.21** (0.03)	−0.09 (0.08)	−0.21 (0.12)
Rapid rehousing	−0.23** (0.01)	−0.21** (0.01)	−0.19** (0.02)	−0.13** (0.04)	−0.18** (0.02)	−0.16** (0.02)	−0.16** (0.03)	−0.12 (0.09)
Other, housing	−0.11** (0.01)	−0.09** (0.01)	−0.13** (0.02)	−0.26** (0.06)	−0.10** (0.02)	−0.08** (0.02)	−0.09* (0.04)	−0.16 (0.11)
Other, non-housing	−0.07** (0.01)	−0.03** (0.01)	0.00 (0.01)	−0.00 (0.03)	−0.06** (0.01)	−0.03 (0.01)	−0.02 (0.02)	−0.13 (0.07)
*Race/ethnicity* (reference group: Non-Hispanic White)								
Non-Hispanic Black	0.05** (0.01)	0.04** (0.01)	0.10** (0.01)	0.10** (0.01)	0.06** (0.01)	0.06** (0.01)	0.09** (0.02)	0.09** (0.02)
*Controls*								
Gender, household structure	X	X	X	X	X	X	X	X
Vulnerabilities/past experiences		X		X		X		X
Vulnerabilities/past X service types				X				X

*Notes*: CoC = continuum of care. All models control for sociodemographics/household structure, continuous age, age-squared, and fixed effects capturing: individuals’ first year of CoC service (2013–2019); service entry date year, date month, and entry date year-month combination. Standard errors are clustered by person. **p < .01. *p < .05 (two-tailed test)

^a^Analytic sample individuals’ age is estimated in 2013, based on birth year.

^b^Type of CoC service-Other, housing (OTH-H) includes: Transitional Housing; Permanent Housing: Housing Only; Permanent Housing: with services but no disability required.

^c^Type of CoC service-Other, non-housing (OTH-N) includes: Coordinated entry; Homelessness Prevention; Services Only; Street Outreach; Other.

Starting with younger adults (25–44), Model 1 suggests that RRH and PSH both exert large, negative effects (15–16 pp) on the probability of returning to CoC services. Net of service type, Black younger adults still exhibit a 4 pp increased risk of returning to CoC services. Race and program type effects among younger adults remain largely stable when a slate of vulnerability and past experience controls are added in (Model 2). The Black–RRH interaction is significant and negative in Model 3; it remains so after including interactions between vulnerability and past experience variables with each CoC service type (Model 4). Overall, RRH is estimated to generate a ~3 pp larger protective effect for younger Black adults versus otherwise-similar White ones.

Across the three older sample strata, PSH and RRH also exhibit large protective main effects for each subgroup relative to the ES counterfactual; these effects are larger for the three older sample strata than they are for the youngest strata. Shifting to examining racial heterogeneity in this program’s effectiveness, PSH exhibits a significantly stronger protective effect among Black individuals in these three groups compared to otherwise-similar White ones. Most striking, PSH exhibits a ~20 pp larger protective effect when comparing Black versus White retirement-age adults. Importantly, this moderation effect does not appear to be explained by subgroup differences in vulnerabilities and past experiences, as we initially predicted.

Supplementary Figure S1, Panel B reports the estimated conditional probabilities of returning to CoC services for White and Black recipients of ES and PSH services based on age- and race-stratified models that include basic control variables (full output available upon request). The figure reinforces the amplified PSH (vs ES) associated drop among Black versus White retirement-age adults.

### Robustness Checks

Additional evidence supporting the enhanced effectiveness of RRH and PSH for Black older adults, particularly retirement-age adults, emerges from several sets of robustness check models. Models that include the same predictors and same age-based stratification structure as Model 4 but employ an event history model, rather than LPM or HLM, specification, reveal the same basic patterns of age and race heterogeneity in PSH and RRH effectiveness reported above; PSH and RRH appear to predict longer delays in returns to service for Black versus White adults within the three older age strata (see Supplementary Table S1). Moreover, hierarchical linear models that nest CoC service spells (level-1) into individual persons (level-2) and adjust standard errors accordingly generate virtually identical results to the LPM models in Table 4 (see Supplementary Table S2).

To probe two potential sources of bias—omitted variable and mortality-induced attrition bias—we return to our LPM specifications from Table 4’s most complete models but subdivide the two older adult groups (ages 55+) into four narrower age strata—55–60, 61–64, 65–68, and 69+—and examine the Black × RRH and Black × PSH interaction terms within each. We believe there is likely to be more balance vis-a-vis observable and unobservable characteristics across race/ethnic groups within each narrowly specified age stratum than there is across the pooled 55–64 and 65+ age groups.

These analyses (presented in Supplementary Table S3) mitigate some concerns regarding mortality-induced attrition and omitted variable bias insofar as the Black × PSH and Black × RRH coefficients are consistently negative across all age-based subgroups. Moreover, there is not a clear pattern of increasing magnitude in the Black × PSH and Black × RRH negative effects as age strata increase, which would be suggestive of mortality-induced attrition. We also run Supplementary Table S3’s age-stratified models with CoC service spells deemed to have resulted in the individual “exiting” to jail (based on HMIS-provided destination codes) removed, because this outcome could also bias our results much in the way mortality could. The results (not shown) are virtually identical to those presented in Supplementary Table S3. Taken together, these results provide additional support for our prediction that “Housing First” interventions, especially PSH, yield larger risk reduction benefits versus ES for older Black adults than for older White adults.

Importantly, however, the most complete models in Table 4 and Supplementary Table S3 suggest that, contrary to our proposed explanation, this heterogeneity pattern does not appear to be explained by Black versus White differences in individual-level vulnerabilities among older adults that PSH and RRH are specifically designed to address. What, then, could explain racial heterogeneity?

## Alternative Explanations for Racial Heterogeneity in “Housing First” Interventions’ Effectiveness: Exploratory Analyses

One reason that PSH and RRH effectiveness may diverge between Black and White older adults is that the two groups systematically sort into different types of PSH and RRH programs that may in turn meaningfully vary in their average effectiveness. For example, if White older adult PSH residents are disproportionately sorted into SS developments, as evidence suggests (Henwood et al., 2024), and these SS contexts are less effective in reducing the risk of returns to homelessness services than are project-based settings (PB) (e.g., due to less comprehensive, consistent service provision), then disentangling the effects of PSH-SS and PSH-PB may “control away” the racial heterogeneity pattern reported above.

The models in Table 5 do just this, splitting PSH into SS and PB components for the full analytic sample (Model 1) and then for each age stratum (Models 2–5). For nearly all age groups, site-based PSH exhibits a much stronger protective effect than does SS PSH for the White reference group. However, the differential effectiveness of SS versus SB PSH does *not* appear to explain the racial heterogeneity pattern among retirement-age adults discussed above. For the full sample (Model 1) and especially for the retirement-age subsample (Model 5), a racial heterogeneity pattern remains detectable vis-a-vis the effectiveness of SS, not PB, PSH.

**Table 5.** Linear Probability Models (Ordinary Least Squares) Predicting Probability of Returning for Additional Los Angeles County CoC Service After Receiving a CoC Service (2013–2019), With PSH Disaggregated by Site-Based Versus Scattered Site

	Model 1: All Ages	Model 2: Age 25–44	Model 3: Age 45–54	Model 4: Age 55–64	Model 5: Age 65+
					
Black × PSH: site-based	−0.03 (0.02)	0.04 (0.04)	−0.03 (0.04)	−0.13** (0.05)	−0.03 (0.11)
Black × PSH: scattered site	−0.12** (0.03)	−0.07 (0.05)	−0.18** (0.05)	−0.03 (0.06)	−0.49** (0.14)
Black × RRH	−0.05** (0.01)	−0.03* (0.01)	−0.06** (0.02)	−0.07** (0.02)	−0.05 (0.04)
Black × OTH-H	0.03* (0.01)	0.07** (0.02)	−0.02 (0.02)	0.01 (0.03)	−0.01 (0.05)
Black × OTH-N	−0.07** (0.01)	−0.06** (0.01)	−0.06** (0.01)	−0.11** (0.01)	−0.04 (0.03)
Black	0.07** (0.00)	0.06** (0.01)	0.06** (0.01)	0.10** (0.01)	0.09** (0.02)
*Focal CoC service: type of service received^a^* (reference group: emergency shelter)					
PSH: Site-based	−0.13** (0.04)	−0.15* (0.06)	−0.10 (0.06)	−0.16* (0.07)	−0.31* (0.14)
PSH: Scattered site	−0.08 (0.04)	−0.09 (0.07)	−0.04 (0.07)	−0.19* (0.08)	−0.20 (0.16)
RRH	−0.07** (0.02)	−0.04 (0.02)	−0.09** (0.03)	−0.13** (0.04)	−0.12 (0.09)
OTH-H	−0.14** (0.02)	−0.11** (0.04)	−0.11* (0.05)	−0.26** (0.06)	−0.16 (0.11)
OTH-N	0.00 (0.01)	−0.00 (0.02)	0.02 (0.02)	−0.00 (0.03)	−0.13 (0.07)
*Age category (as of 2013; ref: 25–44)*					
45–54	0.02** (0.00)				
55–64	0.02** (0.01)				
65+	0.01 (0.01)				
*Controls*					
Gender, household structure	X	X	X	X	X
Vulnerabilities/past experiences	X	X	X	X	X
Vulnerabilities/past × service types	X	X	X	X	X
Person *N*	155,558	81,672	42,747	24,706	6,433
Service *N*	254,061	126,558	74,861	42,786	9,856

*Notes:* CoC = continuum of care; PSH = permanent supportive housing; RRH = rapid rehousing. All models include controls for continuous age (as of 2013) and age-squared, as well as fixed effects capturing: individuals’ first year of CoC service (2013–2019) and service entry date year, service entry date month, and service entry date year-month combination. Standard errors clustered by person. **p < .01. *p < .05 (two-tailed test).

^a^Type of CoC service-PSH = Permanent supportive housing; RRH = Rapid rehousing; OTH-H includes: Transitional Housing; Permanent Housing - Housing Only; Permanent Housing with services, no disability required. OTH-N includes: Coordinated entry; Services Only; Homelessness Prevention; Street Outreach; Other.

A robustness check that replicates Table 5’s model specifications but replaces the return to CoC service outcome with an alternative outcome proxying housing stability—the probability of receiving a post-service HMIS destination code indicating the individual “exited” to renting or owning a housing unit (with or without subsidy) or moving in with family or friends—reinforces this same pattern (see Supplementary Table S4). Retirement-age Black adults who receive PSH-SS see a striking 33 pp increase in the probability of being marked with this promising exit code compared to otherwise-similar White adults who receive PSH-SS. There is no significant Black–White difference in PSH-PB effects on this same outcome.

An exploratory analysis described and presented in Supplementary Material: Methodological Appendix (Section D) provides suggestive evidence for why, on average, Black retirement-age adults who receive PSH-SS see a much larger reduction in risk and boost in probability of exiting to a stable housing situation than do otherwise-similar White retirement-age adults who receive PSH-SS: on average, Black retirement-age adults sort into higher-quality SS programs than do White adults of a similar age. In light of these analyses and past research suggesting the quality of PSH interventions may vary widely (Milburn et al., 2021), we believe racially stratified sorting patterns across PSH programs could partially explain why older Black adults derive larger benefits from PSH in general, and PSH-SS, in particular.

## Discussion and Conclusion

Motivated by the widely documented aging and browning of America’s population, alongside spiraling housing costs within U.S. metropolitan areas, this study examines whether older adults of color exhibit an elevated risk of returning to homelessness services after receiving CoC services and whether promising “Housing First” interventions—namely, PSH and RRH—are especially effective for Black older adults. Using an administrative longitudinal data set tracking all individuals interfacing with CoC services in Los Angeles County, a major epicenter of the homelessness crisis, we advance past work on “Housing First” interventions (Gubits et al., 2018; Henwood et al., 2013) by mobilizing an intersectional approach to evaluate risk and program effectiveness.

Concretely, we reveal an age–race interaction in the risk of return to homelessness services, with risk generally increasing with age, Black adults exhibiting higher risk compared to Whites within all age groups, and the Black–White gap increasing considerably at older ages. Black older adults thus emerge as a particularly high-risk subgroup and clarifying which interventions are particularly effective for them is especially important. Encouragingly, we find that “Housing First” interventions, specifically RRH and PSH, are highly effective overall and associated with even larger risk reductions for older Black adults than for White older adults, when benchmarked against the ES counterfactual.

Clarifying whether—and why—“Housing First” interventions are truly more effective for Black older adults than for White ones constitute important lines of inquiry. Our models suggest that an extensive set of individual-level differences in vulnerability factors and past experiences did not explain this racial heterogeneity pattern. But we cannot discount the possibility that omitted variable bias may underlie it. Our study—like the vast majority of prior homelessness studies, particularly those using HMIS—relies on observational data and thus could not guarantee balance in observable and unobservable characteristics that might contaminate our estimates of RRH and PSH main and interactive effects. Although our extensive robustness checks all suggested limited bias in our core results, experimental and quasiexperimental designs are required to confirm that RRH and PSH effects are truly disproportionate for older adults in general, and Black older adults in particular. Properly powered studies that randomly assign RRH and PSH slots to a large sample diverse in age and race composition would be optimal. Another option might entail leveraging individual-level fixed effects with observational data to ensure group differences in difficult-to-observe individual characteristics do not fully explain PSH and RRH’s estimated effectiveness overall and the programs’ disproportionate effects for key subgroups.

If rigorous designs confirm the age- and race-stratified patterns of RRH and PSH effectiveness we uncovered, future mixed methods studies should further probe racially stratified sorting processes into “Housing First” interventions versus alternative interventions and into specific programs. One sorting-related possibility that could underlie racial heterogeneity in PSH effectiveness is that White older adults who select SS PSH may prioritize neighborhood characteristics over program quality proxies, resulting in exposure to less effective programs. Analyses that account for group disparities in sorting processes and program quality should be applied not only to PSH but also to RRH programs. Space constraints precluded us from deeply probing explanations for racial heterogeneity in the latter’s effectiveness, but our analyses suggest it is a very promising intervention for Black older adults.

More broadly, research and policy should pivot from evaluating the effectiveness of “Housing First” interventions on the general population, which is now solidified (Woodhall-Melnik & Dunn, 2016), to a “precision medicine” approach that theorizes and tests potential axes of heterogeneity—including structural intersectionality, as we do. Analyzing HMIS data offers a promising path to do so, given the data’s statistical power and time horizon, as well as the extensive set of stratifying variables available. Ideally, future researchers will determine how to use HMIS data to assess individuals’ trajectories in ways that expand beyond evaluating whether they returned for additional CoC services. As noted above, this outcome is an imperfect proxy for the risk of returning to homelessness and the risk of other adverse scenarios; for example, if individuals died during or after CoC service, relocated to other CoCs, or became unsheltered homelessness and were not contacted by street outreach, their outcome was marked as a nonreturn. The two latter scenarios may be particularly common among Hispanics who have experienced homelessness, given their elevated rates of undocumented status, which may coincide with higher levels of distrust and lower levels of CoC service engagement.

However, alternative outcomes currently available in HMIS data come with alternative, and likely more severe, sources of bias, including inaccurate service provider data entry. Looking forward, data-sharing agreements between CoCs, encampment clearance programs, eviction courts, health service providers, criminal justice entities, and coroners’ offices may help overcome this key limitation of our work. These agreements may lead to alternative outcome variables that are less susceptible to various sources of bias and more comprehensive by capturing how various CoC services predict risk of unsheltered homelessness (without street outreach), mortality, emergency room visits, criminal justice involvement, or eviction and by enabling more precise coding of outcomes for individuals who move or die after receiving CoC services.

As researchers gain purchase on whether and why certain types of “Housing First” interventions boost various outcomes to a greater extent for some subgroups than for others, policymakers must rapidly expand the number of individuals who receive these services. Although we have provided encouraging evidence regarding these services’ effects for the most vulnerable populations, relatively few people receive RRH or PSH. To avert the looming crisis of housing insecurity and homelessness among older adults, particularly those of color, the capacity of CoCs to deliver high-quality “Housing First” interventions must be rapidly and exponentially increased.

## Supplementary Material

gnaf050_suppl_Supplementary_Material

## Data Availability

The data on which this study is based are restricted access and were obtained from the California Policy Lab under special contractual arrangements to protect sensitive information. The data necessary to reproduce the analyses presented here are not publicly accessible, but interested researchers can set up a data use agreement with the California Policy Lab and Los Angeles Homeless Services Authority.
