# Supplementary material for: Assessing Racial Heterogeneity in “Housing First” Supports’ Effectiveness Among Older Adults Experiencing Homelessness: Evidence From Los Angeles County
Source: Gerontologist. 2025 Feb 6;65(7):gnaf050. doi: 10.1093/geront/gnaf050 (PMC12223365; doi:10.1093/geront/gnaf050)
Supplement: gnaf050_suppl_Supplementary_Material [file gnaf050_suppl_supplementary_material.docx]

**SUPPLEMENTARY MATERIAL**

**METHODOLOGICAL APPENDIX**

**A. Details on Analytic Sample Specification**

As noted in the main text, our analytic sample begins with all HMIS-reported Los Angeles County CoC service contacts tracked within the Research Accelerator dataset that started between 2013 and 2019– a total of 541,831 service contacts. We start with 2013 because prior research suggests there were major data entry lapses among the county’s CoC service providers in earlier years, and HMIS data are derived directly from CoC service providers’ data entry (i.e., service providers input the date of service entry and, if applicable, exit) (Meyer et al., 2023). We stop at 2019 due to concerns that the COVID-19 pandemic yielded substantial changes to CoC data entry quality and CoC service characteristics, including screening criteria and duration of services provided (e.g., eviction moratoria may have prolonged the length of rapid rehousing interventions).

Next, we specify the analytic sample to only include CoC services linked to individuals who were confirmed to be adults (i.e., age 25+, so transition-aged youth are excluded) at baseline (2013), leaving 363,484 service contacts nested within 168,340 adult individuals. Of these service contacts, we remove the 54,788 CoC service records in which an individual either (a) received more than one of the following service types simultaneously– emergency shelter, transitional housing, rapid rehousing, and permanent supportive housing (*N*=2,570)– or (b) received another CoC service that began after the focal CoC service contact entry date but before the focal service contact exit date (*N*=52,218). These two conditions preclude us from disentangling the effects of each specific type of service on our outcome of interest.

Importantly, of the remaining CoC service contacts, we drop the 23,279 contacts marked with entry dates indicating they began less than a week after the entry date or exit date (if applicable; whichever is later) assigned to the individual’s prior CoC service in our 2013-2019 timeframe. The specification helps ensure that high-frequency churning (e.g., moving into and out of a given emergency shelter during the course of a week) does not bias the results. Following a similar logic, we also drop the 1,153 service contacts containing a CoC service entry date within a week of the end of our study period (December 31, 2019).

Finally, to mitigate the possibility of mortality-induced attrition biasing our results, we remove 1,915 of our remaining CoC service records that were marked in HMIS as ending with the death of the service recipient, as well as 28,288 service records where the post-service destination (e.g., unsheltered homelessness, institutional setting) was marked missing. The effect of the former set of service spells on our core outcome of interest– the probability of an individual returning to CoC services– can, of course, not be determined. The missing values for the latter set of service spells raises the possibility that the individual may have died during the course of service, as well.

With these CoC service contacts removed, our final analytic sample includes 254,061 CoC service “spells” nested within 155,558 unique adults.

**B. Control Variables Included in Multivariate Models**

Across all multivariate models, we include fixed effects capturing individuals’ *first year of CoC service* within our timeframe of interest (2014-2019, ref: 2013) and the focal CoC service spell’s HMIS-provided *entry date year*, *entry date month*, and *entry date year-month combination*. We also adjust for continuous age (estimated as of 2013, based on birth year) and age-squared and include binary control variables capturing individuals’ gender identity (female, transgender/nonbinary/other, ref: male) and *household structure* (i.e., whether the individual’s HMIS record indicates she shares a household with another individual).

We include a rich set of explanatory variables to assess whether individual-level vulnerabilities and past experiences– which together proxy the severity of individuals’ risk/acuity– could explain why certain interventions are particularly effective in reducing the risk of returning to homelessness services for certain race/age subgroups. Binary variables capturing vulnerabilities indicate whether the individual is an armed forces *veteran* (versus veteran status-unknown or ref: non-veteran). Dichotomous variables also indicate whether the individual is marked in HMIS data as having each of the following conditions: *physical disability*, *developmental disability*, *chronic disability*, *HIV/AIDS*, *mental disability*, or *substance abuse.*

We adjust for clients’ prior experiences by capturing *the number of distinct CoC service spells between 2013 and 2019* that precede the focal CoC service for a given individual; we also include the square term of this variable to model nonlinearities. Binary indicator variables capture *the number of times the individual has been reported to be homeless in the three years preceding the focal CoC service entry date*: once, twice, three times, four or more times, or an unknown number (ref: not applicable/no times).

**C. Additional Details on Analytic Strategy: Age-Stratified Models**

After assessing age-based heterogeneity in PSH and RRH effectiveness, using Equation 1 in the main text, we stratify the sample across our four age groups (25-44; 45-54; 55-64; 65+) to assess whether there is also evidence racial heterogeneity in these programs’ effectiveness *within each age bracket*. We use the following model across age brackets to do so:

**(Equation 2)
(Received Additional CoC Service After Focal CoC Service)*_ij_ = β_0_* +*β_1_*(PSH)*_ij_* +*β_2_*(RRH)*_ij_* + *β_3_*(OTH-H)*_ij_* +*β_4_*(OTH-N)*_ij_* + *β_5_*(PSH)*_ij_*X(Black)*_j_* +*β_6_*(RRH)*_ij_*X(Black)*_j_* +*β_7_*(OTH-H)*_ij_*X(Black)*_j_* +*β_8_*(OTH-N)*_ij_*X(Black)*_j_* +…+*e_ij_***

Interactions between the binary indicators for each service type and all other race/ethnic groups (ref: non-Hispanic White) are also included in the model, but the focal parameters of interest for these four sets of age-stratified models are ***β_5_*** and ***β_6,_*** which capture how much larger, or smaller, the estimated risk reduction effects of PSH and RRH are (versus the ES counterfactual) for Black adults compared to White adults of a similar age. We expect these coefficients to be significant and negative. If they are, we will run additional models that bring in the individual-level vulnerability and past experience variables described above and interact these variables with the focal CoC service type indicator variables. Attenuation in the Black-PSH or Black-RRH interaction coefficients would suggest that racial differences in individuals’ risk profile may partially explain why RRH and PSH are particularly effective for Black versus White older adults.

**D. Exploratory Analysis of PSH Program Quality**

Recent research on “Housing First” interventions has called for additional attention on program quality differences within the large and heterogeneous group of programs classified as PSH (Milburn et al., 2021). Yet vanishingly few studies have conceptualized how differences in PSH program quality might be properly measured with longitudinal client outcomes data; fewer still have tried to empirically estimate the distribution of PSH program effects using these data. We attempt to do this as part of an exploratory analysis, by drawing on frameworks and approaches within the K-12 school value-added quality literature and by leveraging our large, longitudinal HMIS dataset tracking the characteristics and outcomes of PSH clients before, during, and after, they participate in a PSH program. This exercise may potentially illuminate why Black older adults see considerably larger degrees of risk reduction vis-a-vis returns to homelessness services after entering a PSH scattered site program, compared to otherwise-similar White older adults.

More than two decades of education research has yielded a widely accepted approach to quantifying differences in K-12 school quality: the value-added school effectiveness model. This approach typically leverages longitudinal data and multilevel models (i.e., hierarchical linear models/HLMs) to partition the variance in academic achievement into between- and within-school components, while adjusting for differences in student characteristics that shape achievement but that the school has no, or minimal, control over (e.g., student sociodemographic characteristics; measures of academic achievement prior to entering the school). The *between-*school variance component, estimated after adjusting for student sorting dynamics, is interpreted as the “value-added” effect of a given school on student outcomes. It can be substantively interpreted as how much higher (or lower) the average student would be expected to achieve vis-a-vis a specific outcome (e.g., standardized test scores) if they attended a given school versus the average school (see Lloyd & Schachner, 2021for more details on this approach). (Quasi)experimental research designs have confirmed that this value-added approach using observational data generates school effectiveness estimates that closely approximate estimates that would be recovered from randomized school assignment (Angrist et al., 2017; Deming, 2014).

We believe this approach holds promise for estimating the quality (i.e., value-added effectiveness) of various PSH programs, with quality conceived as a program’s impact on reducing (or increasing) the probability a given individual will return for additional CoC services at some point after entering the PSH intervention. To this end, we isolate the subset of focal CoC services in our analytic sample (pooling adults of all ages) that were marked as a PSH intervention during the 2013-2019 timeframe (CoC service spell *N* = 4,612) and run a three-level HLM with random intercepts and fixed slopes (level-1: CoC service spell; level-2: individual adult; level-3: PSH program identifier) predicting our core outcome of interest: the probability of returning for additional CoC services at some point after receiving the focal PSH service. We include all covariates listed in the Data & Methods section, as well.

Modeling the third-level random intercept in the HLM framework using PSH program identifiers enables us to isolate the portion of the total variance in our outcome attributable to PSH programs, as opposed to individual-level characteristics or temporal dynamics. Further, these random intercepts enable us to quantify residualized program effects (i.e., adjusted for program differences in participant sociodemographics, vulnerabilities, and past experiences) on the risk of returning for additional CoC service for all 288 PSH programs that clients in our analytic sample participated in. These value-added PSH program effects can be substantively interpreted as how much less (or more) likely a given client would be to subsequently return for a CoC service if they attended a given PSH program, compared to the average PSH program.

Our HLM results (detailed model results available upon request) indicate that a one standard deviation increase in PSH value-added program quality decreases the probability of returning to CoC service after beginning the PSH program by six percentage points. We interpret these results as suggesting the 288 PSH programs vary fairly widely in their estimated effects on our core outcome of interest, even after adjusting for selection effects.

Could racial differences in PSH program quality measured in this manner explain why Black older adults see much larger degrees of risk reduction vis-a-vis returns to homelessness services after entering a PSH scattered site program, compared to White older adults? We explore this possibility by comparing the distribution of PSH program value-added estimates for PSH-scattered site programs in which Black older adults in our analytic sample were enrolled compared to the programs White older adults in our sample received.

Among retirement-aged adults (65+) in our analytic sample who participated in a PSH scattered site program, Black adults indeed attended programs with considerably higher levels of estimated value-added effectiveness compared to White adults. Concretely, the average Black PSH scattered site participant age 65+ attended a program estimated to reduce the risk of returning for CoC service by 3pp more than the average PSH program. The average older White PSH scattered site participant attended a program whose value-added effectiveness was about average. In other words, the average older Black adult within a PSH-scattered site setting sorted into a program that was almost half a standard deviation higher in the PSH value-added distribution than was the program accessed by the average older White adult in a PSH-scattered site setting. We thus conclude that Black retirement-age adults may be systematically sorting into higher-quality scattered-site PSH programs than are White retirement-age adults. Our Discussion & Conclusion section explores the implications of this exploratory analysis for future research.

**REFERENCES**

Angrist, J. D., Hull, P. D., Pathak, P. A., & Walters, C. R. (2017). Leveraging Lotteries for School Value-Added: Testing and Estimation*. *The Quarterly Journal of Economics*, *132*(2), 871–919. <https://doi.org/10.1093/qje/qjx001>

Deming, D. J. (2014). Using School Choice Lotteries to Test Measures of School Effectiveness. *American Economic Review*, *104*(5), 406–411. https://doi.org/10.1257/aer.104.5.406

Lloyd, T. & Schachner, J. N. (2021). School effects revisited: The size, stability, and persistence of middle schools’ effects on academic outcomes. *American Educational Research Journal,* *58*(4): 748–84. <https://doi.org/10.3102/0002831220948460>.

Meyer, B. D., Wyse, A., & Corinth, K. (2023). The size and Census coverage of the U.S. homeless population. *Journal of Urban Economics*, *136*, 103559. <https://doi.org/10.1016/j.jue.2023.103559>

Milburn, N. G., Edwards, E., Obermark, D., & Rountree, J. (2021). *Inequity in the Permanent Supportive Housing System in Los Angeles: Scale, Scope and Reasons for Black Residents’ Returns to Homelessness*. California Policy Lab.

**TABLE S1.** Age-Stratified Event History (Proportional Cox, with Hazard Ratios) Models Predicting Risk of Returning for Los Angeles CoC Service after Receiving a Service

|  | **Young Adult (25-44)** (Service *N*=126,558; Person *N*=81,672) | | | | **Middle-Age Adult (45-54)** (Service *N*=74,861; Person *N*=42,747) | | | |
| --- | --- | --- | --- | --- | --- | --- | --- | --- |
|  | Model 1 | Model 2 | Model 3 | Model 4 | Model 5 | Model 6 | Model 7 | Model 8 |
| **Race/ethnicity X CoC service type interactions** | | |  |  |  |  |  |  |
| Black X PSH |  |  | 1.05 (0.11) | 1.02 (0.10) |  |  | 0.76** (0.07) | 0.76** (0.07) |
| Black X RRH |  |  | 0.92 (0.05) | 0.94 (0.05) |  |  | 0.77** (0.05) | 0.80** (0.05) |
| Black X OTH-H |  |  | 1.23** (0.08) | 1.27** (0.08) |  |  | 0.91 (0.06) | 0.93 (0.06) |
| Black X OTH-N |  |  | 0.85** (0.02) | 0.83** (0.02) |  |  | 0.83** (0.02) | 0.83** (0.02) |
| **Focal CoC service: type of service received (ref: emergency shelter)** | | | | |  |  |  |  |
| Perm supportive housing | 0.53** (0.02) | 0.50** (0.02) | 0.50** (0.04) | 0.53** (0.09) | 0.48** (0.02) | 0.48** (0.02) | 0.59** (0.05) | 0.68* (0.11) |
| Rapid rehousing | 0.55** (0.01) | 0.58** (0.01) | 0.57** (0.03) | 0.60** (0.06) | 0.47** (0.01) | 0.48** (0.01) | 0.56** (0.03) | 0.57** (0.07) |
| Other, housing | 0.70** (0.02) | 0.74** (0.02) | 0.64** (0.03) | 0.55** (0.08) | 0.73** (0.02) | 0.76** (0.02) | 0.75** (0.04) | 0.58** (0.10) |
| Other, non-housing | 0.79** (0.01) | 0.93** (0.00) | 0.90** (0.02) | 0.86* (0.05) | 0.80** (0.01) | 0.92** (0.01) | 0.91** (0.02) | 1.02 (0.07) |
| **Race/ethnicity (ref: Non-Hispanic White)** | | | |  |  |  |  |  |
| Non-Hispanic Black | 1.16** (0.02) | 1.15** (0.01) | 1.24** (0.03) | 1.24** (0.02) | 1.14** (0.02) | 1.12** (0.02) | 1.26** (0.03) | 1.24** (0.03) |
| **Controls** |  |  |  |  |  |  |  |  |
| Gender, hhld structure | X | X | X | X | X | X | X | X |
| Vulnerabilities/past experiences | | X |  | X |  | X |  | X |
| Vulnerabilities/past X service types | |  |  | X |  |  |  | X |

|  | **Older Adult (55-64)** (Service *N*=42,786; Person *N*=24,706) | | | | **Retirement-Age Adult (65+)** (Service *N*=9,856; Person *N*=6,433) | | | |  |
| --- | --- | --- | --- | --- | --- | --- | --- | --- | --- |
|  | Model 9 | Model 10 | Model 11 | Model 12 | Model 13 | Model 14 | Model 15 | Model 16 |  |
| **Race/ethnicity X CoC service type interactions** | | |  |  |  |  |  |  |  |
| Black X PSH |  |  | 0.76* (0.10) | 0.75* (0.10) |  |  | 0.53+ (0.19) | 0.64 (0.27) |  |
| Black X RRH |  |  | 0.85* (0.07) | 0.86* (0.07) |  |  | 0.92 (0.14) | 0.97 (0.28) |  |
| Black X OTH-H |  |  | 1.04 (0.09) | 1.04 (0.09) |  |  | 1.01 (0.19) | 1.28 (0.51) |  |
| Black X OTH-N |  |  | 0.71** (0.03) | 0.71** (0.03) |  |  | 0.90 (0.08) | 1.31 (0.29) |  |
| **Focal CoC service: type of service received (ref: emergency shelter)** | | | |  |  |  |  |  |  |
| Perm supportive housing | 0.43** (0.02) | 0.45** (0.02) | 0.53** (0.06) | 0.45** (0.10) | 0.37** (0.06) | 0.38** (0.06) | 0.61+ (0.17) | 0.35** (0.21) |  |
| Rapid rehousing | 0.43** (0.01) | 0.44** (0.01) | 0.47** (0.03) | 0.46** (0.08) | 0.48** (0.03) | 0.51** (0.04) | 0.48** (0.06) | 0.46** (0.17) |  |
| Other, housing | 0.73** (0.03) | 0.76** (0.03) | 0.68** (0.05) | 0.31** (0.07) | 0.72** (0.06) | 0.75** (0.06) | 0.73* (0.11) | 0.52 (0.26) |  |
| Other, non-housing | 0.78** (0.01) | 0.88** (0.02) | 1.00 (0.04) | 0.88 (0.09) | 0.83** (0.04) | 0.92** (0.04) | 0.91 (0.07) | 0.61* (0.14) |  |
| **Race/ethnicity (ref: Non-Hispanic White)** | | | |  |  |  |  |  |  |
| Non-Hispanic Black | 1.19** (0.03) | 1.17** (0.02) | 1.38** (0.04) | 1.35** (0.04) | 1.26** (0.06) | 1.24** (0.05) | 1.33** (0.08) | 1.32** (0.08) |  |
| **Controls** |  |  |  |  |  |  |  |  |  |
| Gender, hhld structure | X | X | X | X | X | X | X | X |  |
| Vulnerabilities/past experiences | | X |  | X |  | X |  | X |  |
| Vulnerabilities/past X service types | |  |  | X |  |  |  | X |  |

**Notes** Analytic sample individuals’ age is estimated in 2013, based on birth year. Type of CoC service-Other, housing (OTH-H) includes: Transitional Housing; Permanent Housing: Housing Only; Permanent Housing: with services but no disability required. Type of CoC service-Other, non-housing (OTH-N) includes: Coordinated entry; Homelessness Prevention; Services Only; Street Outreach; Other. All models control for sociodemographics/household structure, continuous age, age-squared, fixed effects capturing: individuals’ first year of CoC service (2013-2019); service entry date year, date month, and entry date year-month combination. Standard errors are clustered by person. ***p* <.01,**p* <.05, +*p* <0.10 (two-tailed test).

|  | **Young Adult (25-44)** (Level-1 *N*=126,558; Level-2 *N*=81,672) | | | | **Middle-Age Adult (45-54)** (Level-1 *N*=74,861; Level-2 *N*=42,747) | | | |  |  |  |  |
| --- | --- | --- | --- | --- | --- | --- | --- | --- | --- | --- | --- | --- |
|  | Model 1 | Model 2 | Model 3 | Model 4 | Model 5 | Model 6 | Model 7 | Model 8 |  |  |  |  |
| Black X PSH |  |  | 0.01 (0.03) | 0.00 (0.03) |  |  | -0.08** (0.03) | -0.09** (0.03) |  |  |  |  |
| Black X RRH |  |  | -0.03* (0.01) | -0.03* (0.01) |  |  | -0.06** (0.02) | -0.06** (0.03) |  |  |  |  |
| Black X OTH-H |  |  | 0.07** (0.02) | 0.07** (0.02) |  |  | -0.02 (0.02) | -0.02 (0.02) |  |  |  |  |
| Black X OTH-N |  |  | -0.04** (0.01) | -0.06** (0.01) |  |  | -0.05** (0.01) | -0.06** (0.01) |  |  |  |  |
| **Focal CoC service: type of service received (ref: emergency shelter)** | | | | |  |  |  |  |  |  |  |  |
| Perm supportive housing | -0.15** (0.01) | -0.17** (0.01) | -0.17** (0.02) | -0.12** (0.05) | -0.19** (0.01) | -0.19** (0.01) | -0.12** (0.02) | -0.07 (0.05) |  |  |  |  |
| Rapid rehousing | -0.13** (0.00) | -0.13** (0.00) | -0.12** (0.01) | -0.04+ (0.02) | -0.18** (0.01) | -0.19** (0.01) | -0.15** (0.01) | -0.09** (0.03) |  |  |  |  |
| Other, housing | -0.09** (0.01) | -0.08** (0.01) | -0.12** (0.01) | -0.11** (0.04) | -0.09** (0.01) | -0.08** (0.01) | -0.08** (0.02) | -0.11* (0.05) |  |  |  |  |
| Other, non-housing | -0.06** (0.00) | -0.02** (0.00) | -0.03** (0.01) | -0.00 (0.02) | -0.05** (0.00) | -0.02** (0.00) | -0.02** (0.01) | 0.02 (0.02) |  |  |  |  |
| **Race/ethnicity (ref: Non-Hispanic White)** | | | |  |  |  |  |  |  |  |  |  |
| Non-Hispanic Black | 0.04** (0.00) | 0.04** (0.00) | 0.06** (0.01) | 0.06** (0.01) | 0.03** (0.00) | 0.03** (0.00) | 0.06** (0.01) | 0.06** (0.01) |  |  |  |  |
| **Controls** |  |  |  |  |  |  |  |  |  |  |  |  |
| Gender, hhld structure | X | X | X | X | X | X | X | X |  |  |  |  |
| Vulnerabilities/past experiences | | X |  | X |  | X |  | X |  |  |  |  |
| Vulnerabilities/past X service types | |  |  | X |  |  |  | X |  |  |  |  |

**TABLE S2.** Age-Stratified Two-level Hierarchical Linear Models Predicting Probability of Return for Additional Los Angeles CoC Service (2013-2019)

|  | **Older Adult (55-64)** (Level-1 *N*=42,786; Level-2 *N*=24,706) | | | | **Retirement-Age Adult (65+)** (Level-1 *N*=9,856; Level-2 *N*=6,433) | | | |
| --- | --- | --- | --- | --- | --- | --- | --- | --- |
|  | Model 9 | Model 10 | Model 11 | Model 12 | Model 13 | Model 14 | Model 15 | Model 16 |
| Black X PSH |  |  | -0.08* (0.04) | -0.09* (0.04) |  |  | -0.21* (0.09) | -0.18* (0.09) |
| Black X RRH |  |  | -0.06** (0.02) | -0.07** (0.02) |  |  | -0.06 (0.04) | -0.05 (0.04) |
| Black X OTH-H |  |  | 0.01 (0.02) | 0.01 (0.02) |  |  | -0.01 (0.05) | -0.01 (0.05) |
| Black X OTH-N |  |  | -0.10** (0.01) | -0.11** (0.01) |  |  | -0.04+ (0.03) | -0.04 (0.02) |
| **Focal CoC service: type of service received (ref: emergency shelter)** | | | |  |  |  |  |  |
| Perm supportive housing | -0.21** (0.01) | -0.21** (0.02) | -0.15** (0.03) | -0.16** (0.06) | -0.22** (0.03) | -0.21** (0.03) | -0.08 (0.07) | -0.21 (0.14) |
| Rapid rehousing | -0.21** (0.01) | -0.21** (0.01) | -0.18** (0.02) | -0.13** (0.04) | -0.16** (0.02) | -0.16** (0.02) | -0.14** (0.03) | -0.12 (0.09) |
| Other, housing | -0.09** (0.01) | -0.09** (0.01) | -0.11** (0.02) | -0.26** (0.06) | -0.09** (0.02) | -0.08** (0.02) | -0.08* (0.04) | -0.16 (0.11) |
| Other, non-housing | -0.06** (0.01) | -0.03** (0.01) | 0.01 (0.01) | -0.00 (0.03) | -0.05** (0.01) | -0.03 (0.01) | -0.02 (0.02) | -0.13+ (0.07) |
| **Race/ethnicity (ref: Non-Hispanic White)** | | | |  |  |  |  |  |
| Non-Hispanic Black | 0.05** (0.01) | 0.05** (0.01) | 0.10** (0.01) | 0.10** (0.01) | 0.06** (0.01) | 0.06** (0.01) | 0.09** (0.02) | 0.09** (0.02) |
| **Controls** |  |  |  |  |  |  |  |  |
| Gender, hhld structure | X | X | X | X | X | X | X | X |
| Vulnerabilities/past experiences | | X |  | X |  | X |  | X |
| Vulnerabilities/past X service types | |  |  | X |  |  |  | X |

**Notes** Analytic sample individuals’ age is estimated in 2013, based on birth year. Type of CoC service-Other, housing (OTH-H) includes: Transitional Housing; Permanent Housing: Housing Only; Permanent Housing: with services but no disability required. Type of CoC service-Other, non-housing (OTH-N) includes: Coordinated entry; Homelessness Prevention; Services Only; Street Outreach; Other. All models control for sociodemographics/household structure, continuous age, age-squared, fixed effects capturing: individuals’ first year of CoC service (2013-2019); service entry date year, date month, and entry date year-month combination. *** p* <.01,* *p* <.05, + *p* < 0.10 (two-tailed test).

**Table S3.** Age-Stratified Linear Probability Models (Ordinary Least Squares) Predicting Probability of Returning for Additional Los Angeles County CoC Service after Receiving a CoC Service among Older Adults

**Notes** Type of CoC service-PSH: Permanent supportive housing; RRH: Rapid rehousing; OTH-H includes: Transitional Housing; Permanent Housing: Housing Only; Permanent Housing with services no disability required. OTH-N includes: Coordinated entry; Services Only; Homelessness Prevention; Street Outreach; Other. All models include controls for continuous age (as of 2013), age-squared, as well as fixed effects capturing: individuals’ first year of CoC service (2013-19); service entry date year, month, year-month combination. Standard errors are clustered by person. **p <.01, *p <.05, +p<0.10.

|  | Model 1: Age 55-60 | Model 2: Age 61-64 | Model 3: Age 65-68 | Model 4: 69+ |
| --- | --- | --- | --- | --- |
| Black X PSH | -0.09* (0.04) | -0.10  (0.07) | -0.28* (0.13) | -0.14  (0.14) |
| Black X RRH | -0.07**  (0.02) | -0.05  (0.04) | -0.04  (0.05) | -0.05 (0.05) |
| Black X OTH-H | 0.02  (0.03) | 0.01  (0.05) | -0.08 (0.07) | 0.10  (0.08) |
| Black X OTH-N | -0.10**  (0.01) | -0.13**  (0.03) | -0.04  (0.04) | -0.04 (0.04) |
| Black | 0.09**  (0.01) | 0.13**  (0.02) | 0.11**  (0.03) | 0.05+  (0.03) |
| **Focal CoC service: type of service received (ref: emergency shelter)** | | | | |
| PSH | -0.18**  (0.07) | -0.14  (0.13) | -0.32  (0.21) | -0.10  (0.18) |
| RRH | -0.13**  (0.05) | -0.15+ (0.09) | -0.24* (0.12) | -0.03 (0.13) |
| OTH-H | -0.19**  (0.07) | -0.46**  (0.11) | -0.24  (0.15) | -0.10  (0.18) |
| OTH-N | -0.00  (0.04) | 0.00 (0.07) | -0.20* (0.10) | -0.04 (0.10) |
| **Controls** |  |  |  |  |
| Gender, household structure | X | X | X | X |
| Vulnerabilities/past experiences | X | X | X | X |
| Vulnerabilities/past X serv. type | X | X | X | X |
| Person *N* | 18,435 | 6,271 | 3,191 | 3,242 |
| Service *N* | 32,422 | 10,364 | 5,066 | 4,790 |

**Table S4.** Linear Probability Models (Ordinary Least Squares) Predicting Probability of HMIS Destination Code: Rent/Own or Family/Friends after Receiving a CoC Service (2013-2019)

|  | Model 1 | Model 2 | Model 3 | Model 4 | Model 5 |
| --- | --- | --- | --- | --- | --- |
| Age | All | 25-44 | 45-54 | 55-64 | 65+ |
| Black X   PSH: Site-Based | -0.02 (0.02) | -0.06  (0.04) | -0.02 (0.04) | -0.00  (0.05) | 0.04 (0.12) |
| Black X  PSH: Scattered Site | 0.07*  (0.03) | 0.01  (0.05) | 0.15**  (0.05) | 0.02  (0.06) | 0.33**  (0.13) |
| Black X  RRH | 0.02+  (0.01) | -0.00  (0.01) | 0.03+  (0.02) | 0.05**  (0.02) | 0.05  (0.03) |
| Black X  OTH-H | 0.05**  (0.01) | 0.04*  (0.02) | 0.04*  (0.02) | 0.07**  (0.02) | 0.04  (0.05) |
| Black X   OTH-N | 0.02**  (0.00) | 0.02**  (0.00) | 0.02**  (0.01) | 0.03**  (0.01) | -0.00  (0.02) |
|  |  |  |  |  |  |
| Black | 0.02**  (0.00) | 0.02**  (0.00) | 0.02**  (0.00) | 0.01*  (0.01) | 0.02+  (0.01) |
|  |  |  |  |  |  |
| **Focal CoC service: type of service received (ref: emergency shelter)** | | | | |  |
| PSH: Site-Based | 0.23**  (0.04) | 0.18**  (0.06) | 0.25**  (0.06) | 0.32**  (0.08) | 0.37* (0.18) |
| PSH: Scattered Site | 0.19**  (0.04) | 0.10 (0.07) | 0.28** (0.07) | 0.25** (0.09) | 0.15 (0.17) |
| RRH | 0.36**  (0.02) | 0.30** (0.02) | 0.47** (0.03) | 0.50** (0.04) | 0.54** (0.07) |
| OTH-H | 0.38**  (0.02) | 0.32**  (0.04) | 0.41**  (0.05) | 0.47**  (0.06) | 0.45**  (0.10) |
| OTH-N | 0.03**  (0.01) | 0.00 (0.01) | 0.06** (0.02) | 0.05+ (0.02) | 0.13** (0.04) |
|  |  |  |  |  |  |
| **Age Category (as of 2013; ref: 25-44)** | |  |  |  |  |
| 45-54 | 0.00  (0.00) |  |  |  |  |
| 55-64 | 0.01  (0.00) |  |  |  |  |
| 65+ | -0.00  (0.01) |  |  |  |  |
| **Controls** |  |  |  |  |  |
| Gender, hhld structure | X | X | X | X | X |
| Vulnerabilities/  past experiences | X | X | X | X | X |
| Vulnerabilities/past   X service types | X | X | X | X | X |
| Person *N* | 155,558 | 81,672 | 42,747 | 24,706 | 6,433 |
| Service *N* | 254,061 | 126,558 | 74,861 | 42,786 | 9,856 |

**Notes** Type of CoC service-PSH: Permanent supportive housing; RRH: Rapid rehousing; OTH-H includes: Transitional Housing; Permanent Housing: Housing Only; Permanent Housing with services no disability required. OTH-N includes: Coordinated entry; Services Only; Homelessness Prevention; Street Outreach; Other. All models include controls for continuous age (as of 2013) and age-squared, as well as fixed effects capturing: individuals’ first year of CoC service (2013-2019); service entry date year, service month, date year-month combination. Standard errors are clustered by person. ***p*<.01,**p*<.05, +*p*< 0.10 (two-tailed test).

**Figure S1.** Probability of Returning for Additional Los Angeles County CoC Service After Receiving CoC Service (2013-2019)

1. **Unadjusted Probability of Returning for Additional CoC Service by Age and Race**
2. **Conditional Probability of Returning for Additional CoC Service by Age, Race, and Service Type**

|  | **Younger Adult** | | **Middle-Aged Adult** | | **Older Adult** | | **Retirement Age** | |
| --- | --- | --- | --- | --- | --- | --- | --- | --- |
|  | White | Black | White | Black | White | Black | White | Black |
| PSH v. ES Absolute Drop | -18pp | -16pp | -14pp | -23pp | -16pp | -26pp | -9pp | -30pp |
| PSH v. ES Relative Drop | -43% | -33% | -29% | -40% | -35% | -45% | -23% | -60% |

**Notes** Age is estimated based on difference between birth year and 2013. ES stands for Emergency Shelter; PSH stands for Permanent Supportive Housing. Conditional probabilities are based on age- and race-stratified models that include fixed effects for CoC service type, gender and household structure controls, as well as controls for continuous age (as of 2013) and age-squared, as well as fixed effects capturing: individuals’ first year of CoC service (2013-2019); service entry date year, service month, date year-month combination.
